# Developing education materials for caregivers of culturally and linguistically diverse patients: Insights from a qualitative analysis of caregivers' needs, access and understanding of information

**DOI:** 10.1111/hex.12867

**Published:** 2019-02-14

**Authors:** Jamie L. Schaffler, Sarah Tremblay, Andréa M. Laizner, Sylvie Lambert

**Affiliations:** ^1^ Ingram School of Nursing McGill University Montréal Québec Canada; ^2^ McGill University Health Centre Montréal Québec Canada; ^3^ Research Institute of the McGill University Health Centre Montréal Québec Canada; ^4^ St. Mary‘s Research Centre Montréal Québec Canada

**Keywords:** CALD, caregiver, chronic illness, information needs, perceived needs, unmet needs, unperceived needs

## Abstract

**Objectives:**

To explore the information needs of caregivers of culturally and linguistically diverse (CALD) patients, and how they access and understand health information related to the management of their care person's chronic illness(es).

**Background:**

Caregivers of CALD patients experience greater unmet needs compared to the general caregiver population. They experience many challenges in identifying resources and accessing formal supports to aid in self‐management behaviours.

**Methods:**

Eleven caregivers were recruited from outpatient clinics in Québec, Canada. Consenting caregivers participated in one face‐to‐face or phone interview. A qualitative descriptive design and inductive content analysis were used to identify themes.

**Results:**

Caregivers described a “village” approach to caregiving in which more than one individual was involved in patient care. The specific roles ascribed to caregivers defined their information needs. Caregivers described two categories of information needs: perceived and unperceived. Perceived information needs were explicit, and centred on the medical management of illnesses. Unperceived needs were unrecognized knowledge gaps that emerged during interviews and focused on self‐care.

**Conclusion:**

Although caregivers' perceived needs are often met, their unperceived needs remain unmet. Health‐care providers should perform need assessments to identify caregivers' unperceived needs, with the aims of providing culturally competent care and ongoing support.

## 1 BACKGROUND

As many as 60% of Canadians are diagnosed with a chronic illness, many of whom are from a culturally and linguistically diverse (CALD) background.^1^ Culturally and linguistically diverse refers to individuals who are foreign born, whose main language spoken at home is neither English nor French, who are proficient in neither English nor French, and have non‐Indigenous status.^2^ Culturally and linguistically diverse populations include both immigrants as well as persons born outside of Canada with Canadian citizenship. Indigenous persons are excluded due to their unique experiences and needs as first nation people, which differ significantly from other minority groups.^3^

In Canada, 20.6% of the population is foreign‐born. One concern is that the incidence of chronic illness is higher among CALD populations compared to the general population.^4^ Despite this, access and usage of health‐care services among CALD populations are low.^5^ This may be related to the unique challenges faced by these communities including unfamiliarity with the health‐care system, a lack of culturally and linguistically appropriate information, and being unable to communicate effectively with the treating team.^6^ These barriers may also in part account for the findings that CALD individuals often have a poor understanding of their condition and associated medical treatments.^4^

Living with a chronic illness requires that patients engage in self‐management activities to enhance health outcomes such as symptom management, pain control and role functioning.^7^ Adams et al^8^ described self‐management as the tasks that an individual must undertake to live well with one or more chronic conditions. To engage effectively in self‐management, patients must develop five core skills, including problem‐solving, action planning, decision making, locating and utilizing resources and forming partnerships with health providers.^9^ These skills facilitate the medical, emotional and role management of chronic illness,^9^ which in turn leads to improved health.^10^ Nonetheless, CALD patients face significant barriers to engaging in self‐management, as they lack the necessary skills, knowledge, and support required,^6^ potentially compromising their overall health and well‐being.

Given the significant burden of chronic illnesses, CALD patients often rely on family or informal caregivers to help in illness self‐management. Studies have found that 80% of individuals diagnosed with chronic illness receive informal care from family caregivers.^11^ Nonetheless, caregivers often report feeling unprepared to take on illness management tasks, and lacking formal support from health‐care providers.^12^ As a result, research has demonstrated high levels of anxiety and depression, and poorer quality of life (QoL) for both the caregiver and patient.^12^, ^13^ As the burden of care is even higher among caregivers of CALD patients,^14^ greater anxiety and depression, overall poorer QoL, and greater unmet needs have been reported by caregivers of CALD patients in comparison with the general caregiving population.^14^, ^15^, ^16^ The particular vulnerability of these caregivers might be explained in part by: (a) the cultural and social expectations of care (eg, familial obligations), (b) barriers to health‐seeking behaviours (eg, limited health literacy) and (c) a lack of support from health‐care providers.^17^ In addition, caregiver characteristics such as familism, levels of acculturation, service barriers, and cultural beliefs and values may account for the differential experience of this sub‐group.^18^ Research has shown that caregivers of patients from CALD backgrounds may be less likely to use formal supports to access health information, or may be unable to navigate within the health‐care system to identify supportive resources.^15^

Despite strong evidence of burden, the information needs of caregivers of CALD patients remains largely under researched.^19^, ^20^ Therefore, the research questions this study addresses are: (a) What are the information needs of caregivers of patients diagnosed with chronic illness(es) from CALD backgrounds; (b) How do caregivers of CALD patients access and understand health information; and (c) What do caregivers of CALD patients think of available instructional materials?

## 2 METHODS

### 2.1 Design

A qualitative descriptive design was used, as this approach attempts to explore the “subjective nature of social reality” by “providing insight from the perspective of participants,” and is particularly useful in understanding the experiences of people living with chronic illnesses.^21^ The research ethics committees of the university‐affiliated hospitals approved this study.

### 2.2 Recruitment

**Figure 1:**
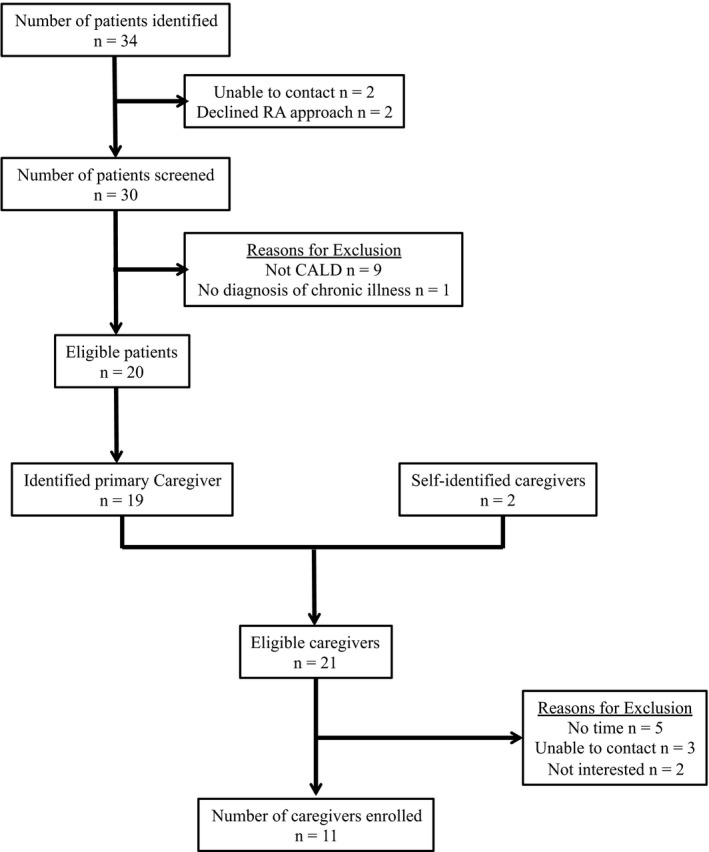
Caregivers' response rate throughout the recruitment and interviewing process

Using convenience sampling, 11 caregivers of CALD patients were recruited (Figure 1). Caregivers were eligible to participate if they provided care to someone who did not speak English or French (as their primary language), was born outside of Canada, was diagnosed with a chronic physical illness, and was being cared for as an outpatient at one of the participating hospitals. Caregivers were eligible regardless of whether or not they themselves were CALD, as many informal caregivers are second‐generation immigrants.^22^ Thus, their upbringing is dually rooted in Canadian culture as well as their distinct cultural identity. Participants were excluded if: (a) they lived more than 40 km away from hospital; (b) the care recipient was diagnosed with a psychiatric disorder, in the absence of a chronic physical illness; (c) the care recipient was deemed too medically unstable; and (d) the caregiver was providing care to a paediatric patient. Individual interviews were favoured (as opposed to dyadic interviews) as this allowed for a more open discussion of caregivers' information needs rooted in the specific challenges encountered while caring for their chronically ill counterparts.^23^

Caregivers were mainly recruited from outpatient clinics across three university‐affiliated hospitals in Québec, Canada. Eligible caregivers were first approached by a member of their treating team who asked about their interest in participating in the research study. Caregivers could also self‐refer to the study through one community support organization advertising the study through their listserv. Interested caregivers were then introduced to a member of the research team who provided additional information about the study and reviewed study materials.

### 2.3 Data collection

Caregivers who provided written consent participated in one interview in their preferred language. The first two authors or a research assistant conducted the interviews. Nine interviews were conducted in English, one in French, and another in Greek using a phone interpretation service. Interviews took place at the hospital, in participants' homes or over the phone. Interviews were audio‐recorded and ranged from 30 to 71 minutes ($\overset{¯}{x}$ = 52.3). All interviews followed a semi‐structured interview guide to explore: (a) the challenges or difficulties encountered by the caregiver; (b) the influence of the caregiving role on the participant; (c) how the caregiver learned to manage their care recipient's illness(es); and (d) the caregivers' information needs. Finally, caregivers were shown Coping‐Together—a self‐directed instructional material developed to promote “adaptive, individual and dyadic coping skills” for chronic illness management^24^ or a generic caregiver information booklet. These resources served to prompt further discussion of caregivers' needs and obtain their opinions of the materials. Electronic copies of the instructional materials were available by email for participants who were interviewed by phone. Finally, all participants completed a socio‐demographic questionnaire.

### 2.4 Data analysis

**Figure 2:**
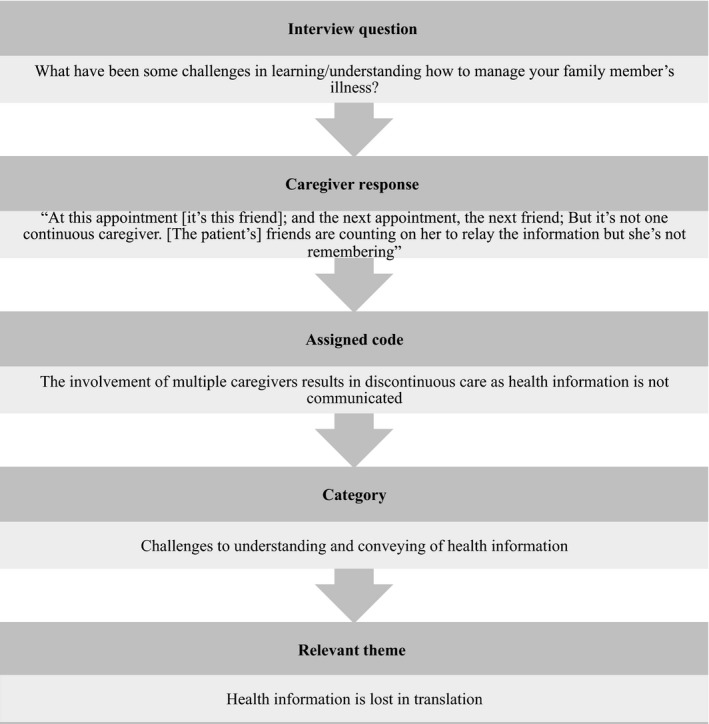
Sample of the coding process using an inductive content analysis approach

Interviews were transcribed verbatim and translated into English by a translator as needed (n = 1, Greek). The French transcript was not translated as all investigators had working knowledge of French. All interviews were analysed in English using an inductive content analysis approach to achieve thematic saturation.^25^ This involved reading all transcripts several times to gain an understanding of the main ideas presented by participants. While the authors recognize that it is best to analyse transcripts in their original language, high quality results have been produced with translation.^26^, ^27^ The transcripts were then analysed using open coding. A list of codes, reflecting the participants' own words, was generated from a line‐by‐line thematic analysis of the data.^25^, ^28^ Codes were words or short sentences that described the essence of the excerpt. The next phase of coding involved classification of similar codes into meaningful categories illustrating the caregivers' information needs, access and understanding.^25^, ^28^ The categories were then grouped into clusters to identify themes, which were representative of the researchers' understandings of caregivers' narratives.^29^ All members of the research team discussed the themes and came to a consensus.^25^ An example of the coding process is shown in Figure 2. Participants' socio‐demographic questionnaires were analysed using descriptive statistics in Excel version 14.7.0.

### 2.5 Trustworthiness

Trustworthiness was achieved through credibility, dependability, confirmability and transferability.^30^ Credibility was achieved through the interview process, by eliciting participant feedback on emerging categories and themes, and ongoing peer debriefing amongst the research team.^30^ Transcript credibility was achieved through independently transcribing and spot‐checking transcripts.^26^ Dependability was achieved through stepwise replication of primary data coding by at least two members of the research team for the first seven transcripts^31^, ^32^ and the iterative process of revising the interview guide to reflect emerging findings. An audit trail of the research process and all decisions made was kept.^31^, ^32^ Finally, transferability was addressed by providing rich descriptions of findings.^31^

## 3 RESULTS

### 3.1 Study participants

Table 1 provides a detailed description of the demographic characteristics of the eleven participants. Caregivers were predominately female (n = 9) and ranged in age from 20 to 78 years ($\overset{¯}{x}$ = 52.3 years). Patients were mostly cared for by their adult children (n = 8) and many caregivers were neither foreign‐born (n = 5) nor restricted in their language proficiencies in English or French (n = 9). As such, only two caregivers were considered to be CALD themselves. The caregivers who participated represented a diverse cultural population including Italian (n = 5), Chinese (n = 2), Greek (n = 2), Romanian (n = 1) and Vietnamese (n = 1) cultures.

**Table 1:** Participant characteristics

Characteristics (n = 11)	n (%)		
Age (years)			
≤20	1 (9.0)	Range	20‐78
21‐40	2 (18.2)	Mean ± SD	52.3 ± 20.6
41‐60	4 (36.4)	Median	52.0
61‐80	4 (36.4)		
Gender			
Female	9 (81.8)		
Male	2 (18.2)		
Relationship to patient			
Spouse	3 (27.3)		
Adult child	8 (72.7)		
Caregiver background			
Non‐CALD	9 (81.8)		
CALD	2 (18.2)		
Education completed			
Elementary	2 (18.2)		
Secondary (high‐school)	5 (45.4)		
College/University degree	4 (36.4)		
Employment			
Full time	4 (36.4)		
Part time	2 (18.2)		
Retired	5 (45.4)		
Household income			
$20 000 to $39 999	2 (18.2)		
$40 000 to $59 999	1 (9.0)		
$60 000 to $79 999	0 (0.0)		
$80 000 to $99 999	4 (36.4)		
≥$100 000	1 (9.0)		
Missing data	3 (27.4)		
Primary diagnoses		Multi‐morbidities	n = 8 (72.7%)
Renal	4 (36.4)		
Endocrine	2 (18.2)		
Musculoskeletal	2 (18.2)		
Cancer	2 (18.2)		
Neurological	1 (9.0)		

CALD, Culturally and linguistically diverse.

### 3.2 Determinants of information needs

**Figure 3:**
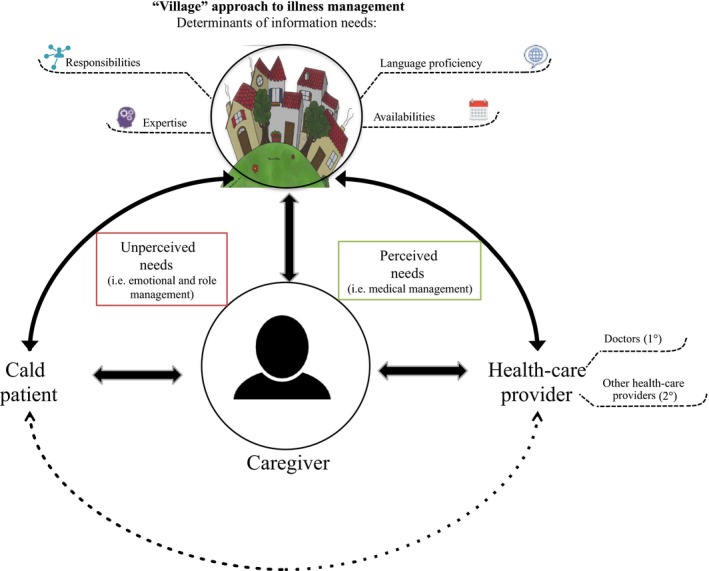
Summary of key findings. Caregivers act as information gatekeepers by transmitting information via direct communication (solid lines) with the patient, village and health‐care providers. Communication between the culturally and linguistically diverse patient and the health‐care provider is indirect (dotted line)

The key findings are summarized in Figure 3. Caregivers' information needs appeared to be determined by what was described as a “family or community approach” to caregiving, and the subsequent responsibilities performed by caregivers within that “community.” Almost all caregivers in this study (n = 10) reported that, although they were the primary caregiver, they relied heavily on other family members. Adult children as caregivers relied principally on their siblings (n = 7), extended family (n = 4) and/or their other parent (n = 4). Conversely, spouse caregivers (n = 3) relied exclusively on their adult children. Therefore, patients had up to six caregivers. For caregivers, this “community” approach to illness management seemed to allow for the division of responsibilities among the caregivers and contributed positively to the sustainability of their role. One caregiver explained: “My sister and brother will be around, and my sister‐in‐law. […] That makes it easier […] We're really a village. That's what it takes” (CG3003). This “village” is represented at the top of Figure 3. The roles and responsibilities assigned to each member of “village” represent the first determinant of caregivers' respective information needs.

An additional three determinants of caregivers' information needs were identified (see Figure 3). The first determinant was caregivers' proficiency in English or French. Although caregivers described a range of roles and responsibilities (summarized in Table 2), tasks that seemed to arise from the CALD context were as follows: (a) “translation” (n = 11); (b) “communication” (n = 8) and “coordination” (n = 9) of care among the village; (c) dealing with the patient's “insecurities” about health information provided to him or her by health‐care providers (n = 5); (d) accessing health information (n = 5); (e) “confirming” health information (n = 3); and (f) dealing with the cultural stigma of illness(es) (n = 3). One caregiver said: “Because she doesn't speak English or French, the social worker said […] ‘I have to talk with her […] you have to be there to translate” (CG4001). As the caregivers were proficient in English or French, they became indispensable to establishing communication between the health‐care provider and patient. The centrality of the caregivers' role in establishing the patient and health‐care professional relationship is illustrated in Figure 3 by having the caregiver in the middle. However, when caregivers themselves were CALD (n = 2), they experienced challenges in fulfilling this role.

**Table 2:** Responsibilities of caregivers (n = 11)

Domain	Specific responsibilities	n (%)
“*TAKING CARE” OF THE* “*MEDICAL THINGS*”		
Illness management	Medication preparation and administration	2 (18.2)
	Monitoring signs and symptoms	6 (54.5)
	Monitoring treatment adherence	6 (54.5)
	Monitoring side‐effects of treatment	6 (54.5)
	Monitoring treatment efficacy	5 (45.4)
	Monitoring functional abilities	2 (18.2)
	Accessing health information^a^	5 (45.4)
	Confirming health information^a^	3 (27.3)
	Surveillance (eg, watching patient at appointments)	4 (36.4)
Communication	With patient* (eg, translation, repeating information)	11 (100)
	Communication within caregiving network^a^	8 (72.3)
	Communication with health‐care team^a^	8 (72.3)
“TRYING TO *BALANCE*” LIFE ROLES		
Time management	Of individual schedule (“manage my own time”)	9 (81.8)
	Across caregiver network (“coordinating our schedules”)^a^	9 (81.8)
Household tasks	Cooking & cleaning	4 (36.4)
	Managing finances	3 (27.3)
	Running errands for patient	1 (9.0)
Help with Activities of daily living (ADLs)	Transportation	4 (36.4)
	Dressing	2 (18.2)
	Bathing	2 (18.2)
	Mobility	2 (18.2)
Seeking resources	Accessing and advocating for resources	4 (36.4)
“BEING STRONG ENOUGH TO HANDLE” THE ILLNESS		
Providing support to patient	Accompaniment	8 (72.3)
	Dealing with stigma of illness^a^	3 (27.3)
	Helping patient maintain relationships	1 (9.0)
Managing patient's emotions about illness	Insecurities related to health information provided^a^	5 (45.4)
	Frustration	4 (36.4)
	Loss of control	2 (18.2)
	Fear	2 (18.2)
	Anxiety	2 (18.2)
	Depression	2 (18.2)
	Feeling unsupported	1 (9.0)
Managing own emotions about illness and/or caregiving role	Concern for patient's well‐being	8 (72.3)
	Stress	5 (45.4)
	Reaction to diagnosis	5 (45.4)
	Frustration	5 (45.4)
	Fear	4 (36.4)
	Guilt	4 (36.4)
	Adapting to change to one's own life	2 (18.2)
	Feeling unsupported	2 (18.2)

a Denotes responsibilities that appear to be a consequence of the patient's limited proficiency in English or French and/or the “network/village” approach to caregiving.

The second determinant was cultural expectations surrounding the provision of care: “In the Italian culture that's the way we are. They take care of us when we're young; we take care of them when they get old” (CG3002). As many caregivers also experienced competing social role demands (eg, work and parenting), they had to engage in role management both as an individual caregiver and across the village: “We assign each other certain things. You know: ‘Can you go, because I can't'. At the end of the day, […] we have obligations to respect” (CG3003).

Expertise was the last determinant described by caregivers and determined the division of responsibilities within the village. In support, one caregiver explained: “I'm an OT by training and my sister was a social worker. So we always took care of the psychosocial, medical and my other siblings take care of the financial aspect. They're both business people” (CG3003). Caregivers (n = 4) also gained expertise through an “ongoing process” (CG2001) of “day‐to‐day learning,” facilitated by observing the patient, the health‐care providers, or other patient‐caregiver dyads and asking questions.

### 3.3 Information needs

**Figure 4:**
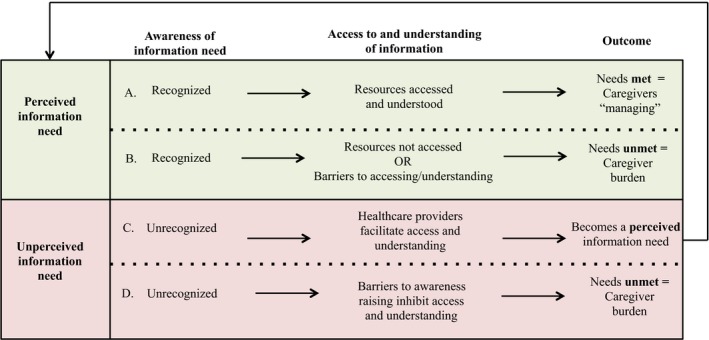
Pattern of information needs, access and understanding among caregivers. This figure depicts the four patterns of information needs, access and understanding among caregivers of CALD patients diagnosed with a chronic illness

As identified in Figure 3, based on their responsibilities, caregivers described two categories of information needs: perceived and unperceived. These needs were further categorized as being met or unmet according to their level of access and understanding of information. This categorization resulted in four patterns of information needs, access and understanding, which are summarized in Figure 4.

#### 3.3.1 Perceived information needs

Perceived information needs were those needs that caregivers explicitly stated when asked directly to identify topics for which they required additional information. The perceived information needs reported by the majority of caregivers (n = 9) centred on the “medical things” such as “knowing more about the disease” (n = 3), adhering to illness‐specific dietary requirements (n = 6), anticipating changes in health status (n = 5), managing emergency situations (n = 1), understanding treatment risks (n = 2) and accessing financial assistance for illness management (n = 2). In support, one caregiver stated: “I would rather have information on my Dad's illness that can tell us what my dad needs to avoid, and what is good for him. For example, in terms of food… then at least we know what to do” (CG1002). Another caregiver who was tasked with accompanying her mother for dialysis treatments explained: “I didn't know what [dialysis] entailed. After researching it, I found it was scary. I didn't have the knowledge” (CG2002).

Nine caregivers spoke of actively searching for answers to their perceived information needs. These needs were met in one of three ways: caregivers independently sought out information (n = 2), a member of the health‐care team provided the information (n = 2) or a combination of these (n = 6). When caregivers' perceived needs were met, it allowed them to engage in “ongoing” illness management by “taking it one day at time,” developing a “routine,” “doing what [they had] to do” or “taking things as they come.” As indicated in Figure 4A, in these instances the individual caregivers and the “village” felt as though they were “managing.”

Of these nine caregivers, six reported barriers to accessing information and/or resources to meet their perceived needs. Identified barriers included having to work during treatments (n = 1) or hospital‐led information sessions (n = 1), lack of communication within the village (n = 1), the patient's ineligibility for programmes due to non‐Canadian citizenship and language barriers (n = 1) and a lack of culturally and linguistically tailored home services (n = 2). One caregiver explained: “[The hospital] sent a letter saying that they were having a day [where] they would explain [dialysis], but I was working at that time” (CG2002). Those caregivers who were unable to overcome these barriers expressed dissatisfaction with their caregiver role (see Figure 4B).

#### 3.3.2 Unperceived information needs

Unperceived needs were unrecognized knowledge gaps, and thus were not initially identified by caregivers (see Figure 4C,D). These needs typically emerged as awareness was raised during the interviews. As a result, caregivers were not actively engaged in acquiring information to fill these gaps. Unperceived needs included: (a) managing personal and patient emotions (n = 9); (b) self‐care strategies such as stress management (n = 8); (c) accessing resources (n = 3); (d) finding time for oneself (n = 3); and (e) skills training on time management (n = 6) and decision making (n = 2). For example, near the end of the interview, one caregiver said: “You know what, you just helped me by talking out loud to define what it is I need. I need just couple of hours or a couple of times a week, to have somebody to stay with [my] mother” (CG4001). For all of these caregivers, it was the first time they discussed their unperceived needs.

Most caregivers (n = 10) reported that they had never received caregiver‐specific materials. Three of the nine caregivers who identified unperceived needs had a preference not to address them. For example, one caregiver stated: “Like an ostrich, I bury my head in the sand […] I don't know what to know about it. I don't want to say ignorance is bliss, but I don't think we can prepare for what the future brings.” Some caregivers did not see the benefits of caregiver resources (n = 4). However, upon review of offered materials addressing unperceived needs, all caregivers found the information to be helpful. Therefore, not only are unperceived needs not recognized by caregivers, most caregivers did not recognize the benefit of addressing these until the material was reviewed with them. Figure 4D illustrates how such barriers to the process of “awareness raising” of information needs seemed to increase the burden of care.

### 3.4 Sources of health information accessed

Caregivers accessed health information from three main sources to address their perceived needs: interpersonal, paper‐based and technology‐based sources.

#### 3.4.1 Interpersonal source: health‐care providers

As indicated in Figure 3, caregivers received health information to meet their perceived needs predominately from doctors (n = 8). Secondary sources of health information were accessed when doctors were unavailable and included nurses (n = 7), pharmacists (n = 4), social workers (n = 1), physiotherapists (n = 1), dieticians (n = 2) and community resources such as clinics (n = 2). Nurses were often perceived as a source of information due to their proximity to doctors (n = 4). Likewise, caregivers described using nurses to gain access to the doctor as they “can identify when the doctor will be by,” they “can just talk to the doctor,” or they “work with [the doctor].”

When discussing sources of health information, caregivers reported different levels of success in accessing health information and services from health‐care providers based on three dimensions: availability, accessibility and accountability. Availability referred to the ability of the health‐care provider to take time to speak with the caregiver and patient, to answer their questions and provide health information. Available providers were said to be more helpful to caregivers in accessing and understanding health information than those who were unavailable.

Accessibility dealt with how easily caregivers could contact health‐care providers. Caregivers of patients' who had more frequent contact with health‐care providers spoke of being more satisfied with their encounters. This seemed to improve caregivers' confidence in accessing information: “The good thing about him being in dialysis is that nurse[s] know him. Even though they don't understand what [he's] saying, they [have learned to] know what he wants. Most importantly, [because] they know my Dad, I feel more confident asking them” (CG1002). Conversely, transient contact with providers, left caregivers feeling dissatisfied with their family member's care.

Finally, caregivers (n = 3) described accountability as a health‐care provider's ability to carry out illness‐specific responsibilities that facilitated caregivers' provision of medical care. One caregiver spoke of a pharmacist who helped her in managing her mother's medications: “They have it prepared […] they're all labeled perfectly, whether it's morning, lunch, afternoon, or before bed […] whereas if you have [the medications] in vials, it's very difficult to know if [she's] taken them or not” (CG2002). Conversely, another caregiver spoke of her difficulties in accessing needed services: “I thought that we settled it, every Wednesday 12 o'clock. So I [went] there at quarter to twelve to make sure, and nobody showed up […] I got upset and I called [the social worker]. I said: ‘what is this?'” (CG4001).

#### 3.4.2 Paper‐based sources: pamphlets

Five of the eleven caregivers reported using pamphlets received from health‐care providers to address their perceived information needs.

#### 3.4.3 Technology‐based sources: internet

Five caregivers accessed the Internet to obtain health information. Those who expressed not using the Internet (n = 3) said it was because of a lack of trust in online materials. No caregivers reported using online support groups or social media.

### 3.5 Lost in translation: challenges to understanding and conveying health information

Seven caregivers described facing challenges in understanding and/or conveying health information. Caregivers reported that various members of the village (themselves included) experienced difficulties understanding health information due to a lack of familiarity with medical terminology (even if they spoke English or French), and/or a lack of understanding of medical information. As stated by one caregiver: “Sometimes the only challenge [I face] in learning his illness is trying to understand terms” (CG1002).

Caregivers also spoke of challenges conveying health information to their care recipient, and across the village. For example, one caregiver spoke of difficulties in translating medical information into the patient's native language: “There's like a really huge off, from what I say, or express, or explain, compared to the […] information itself” (CG1002). Communication was described as challenging for some caregiving “villages” mainly as a result of the discontinuous nature of care provided: “At this appointment [it's this friend]; and the next appointment, the next friend; But it's not one continuous caregiver. [The patient's] friends are counting on her to relay the information but she's not remembering” (CG2001).

### 3.6 Caregivers' recommendations for future instructional materials

Almost all caregivers in this study reviewed instructional materials on topics such as symptom management (n = 2), making treatment decisions (n = 1), dealing with stress (n = 2), supporting oneself and one's care recipient (n = 3) and getting support from health‐care providers (n = 1). All caregivers who reviewed the Coping‐Together materials reported that these were helpful in meeting both their perceived and unperceived needs. Further, they provided suggestions for how these materials should be formatted and disseminated to better address their needs. Table 3 provides a summary of the recommendations pertaining to the ideal length, presentation, language and dissemination of resources. As caregivers reviewed the materials, they emphasized that having easy access to the information was critical, and that the information needed to be practical, including specific strategies to facilitate the provision of care. They emphasized that health‐care providers should distribute materials to supplement their verbal explanations, with particular consideration to the patient's stage along the illness trajectory.

**Table 3:** Desired characteristics of information materials for caregivers

Element	Summary of recommendations	Supporting data (n = 8)
Length of materials	Caregivers preferred materials that were comprehensive, regardless of length, so long as all the information was relevant	“Even though it's a bit big…I think it's good because at least [its] got information we need” (CG1002)
Presentation of materials	Caregivers emphasized useful elements of materials such as breaking up information into smaller chunks, providing an overview of included topics and where they are found within the material, and including specific strategies to deal with their concerns	“There's a lot of good information but maybe if it was broken up in smaller pamphlets then it might be easier to digest” (CG3003) “There's a lot of little tips like if this happens, you have to do this, this, this and this. I find that would be very helpful” (CG2002)
Language of materials	Some caregivers (n = 3) reported preferring information in their native language, and others (n = 4) reported that having it offered in English or French was sufficient	“Even though I'm having to explain some of the jargon, at least she's reading it and it's in her own language. So that would definitely facilitate things” (CG3003)
Who should distribute materials?	Caregivers preferred to receive information from health‐care providers, notably doctors	“The doctor should be able to refer or to say that it's in my waiting room you can pick up this” (CG4001) “That's the kind of book that they should give in the beginning when you go at the [healthcare] center” (CG2004)
How should materials be distributed?	Caregivers (n = 2) spoke of the importance supplementing written materials with education by a health‐care provider	“This is something you have to go through even with a doctor […] It's going to tell you what to look for… But it's not really going to give you an answer exactly” (CG3002)
When should materials be distributed?	The findings on the preferred timing to receive materials were mixed. Information should be received close to the time of diagnosis (n = 2) to facilitate understanding and the provision of care, while also considering the caregiver's readiness to receiving information (n = 2)	“There's more problems in the beginning […]. I think as soon as someone is diagnosed, I think is a perfect time” (CG2002) “It would have been too much I think in the beginning. I would have to say towards the middle […]The first time I was there I just didn't want to be there” (CG2004)

## 4 DISCUSSION

Although caregivers of CALD patients have been identified as a particularly vulnerable group because of their high risk of burden, their needs remain understudied.^14^, ^15^ To our knowledge, this is the first qualitative study describing the information needs of caregivers of CALD patients diagnosed with a range of chronic illnesses, and how they access and understand health information to care for a family member. Also, this is the first study gaining insight into the preferences for education materials of caregivers' of CALD patients.

The first key finding of the study is that caregivers of CALD patients reported information needs that transcend ethnicity and were akin to the self‐management domains proposed by Lorig, Holman^9^: (a) taking care of the medical things or medical management, (b) trying to balance life roles or life roles management and (c) being strong enough to handle the illness for themselves and their care recipient or emotional management. Of these, caregivers were most able to articulate their medical information needs (ie, perceived needs), whereas those pertaining to role and emotional management were identified throughout the interview process (ie, unperceived needs). This finding corroborates those of other studies among non‐CALD caregivers and patients with chronic illnesses^33^, ^34^

Although there have been many studies documenting non‐CALD caregivers' perceived needs, much less has been written on the unperceived needs of CALD and non‐CALD patients and their caregivers.^35^, ^36^ One related concept that has received some attention in the literature is unmet needs. Unmet needs are defined as “needs that are not addressed and where additional support is required”.^37^ Most studies have identified that the highest prevalence of unmet needs lie in the emotional and psychological domains (ie, unperceived needs).^33^, ^38^ This suggests that all caregivers may have unperceived needs, and it is only through explicit assessments that these needs are brought to their attention. Other reasons that caregivers might continue to experience unmet needs include the following: non‐disclosure of their personal needs believing that the care recipient's needs are more important; time constraints or insufficient knowledge of resources among health‐care providers; and a lack of acknowledgement of the caregiver as a patient.^38^, ^39^

Despite the similarities in information needs among caregivers of non‐CALD and CALD patients, unique information needs were also identified. These were: locating culturally and linguistically specific resources for the patient, and approaches to facilitate the transmission of information within the caregiving “village.” Caregivers described that these needs were determined, in part, by the village approach to caregiving. As this is the first study of its kind, there is no literature to compare this against; however, studies among CALD patients have found ethno‐specific illness management needs.^2^, ^4^ In support, a recent study identified that Chinese patients had a unique need pertaining to traditional Chinese medicine.^2^ Further, Muslim patients have emphasized that treatment plans for diabetes should integrate the obligation to fast during Ramadan.^4^ The benefits of providing access to ethno‐specific resources are widespread, with CALD communities reporting positive outcomes such as personalized care, improved clinician relationships and improved self‐efficacy in accessing health‐care services.^40^

A second key finding of the current study was that caregivers of CALD patients played an intermediary role as information gatekeepers, requiring them to have an in‐depth understanding of the illness. This role required that caregivers bridge communication between the patient, the health‐care providers and the village to ensure that the information needs of all stakeholders were met. In support, cross‐cultural research suggests that health‐care providers highly value caregivers of CALD patients as they function to: (a) bridge inequalities that arise from limited health literacy and language proficiencies; (b) provide information on symptoms and treatment side‐effects that the patient is unable to communicate; and (c) allow for the coordination of ongoing contacts with health‐care services.^17^, ^41^ In taking on the role of information gatekeeper, caregivers in the present study described needing high levels of health literacy, self‐efficacy and strong communication skills, which resonates with other CALD studies.^42^, ^43^

Although studies suggest that non‐CALD caregivers can fulfil a similar role of information management,^44^, ^45^ what appears to be unique is the greater intensity of this role among caregivers of CALD patients. This likely relates to the linguistic mismatch between patients and health‐care providers, thus increasing the reliance on caregivers to fill knowledge gaps. Caregivers' of non‐CALD patients are often referred to as a hidden workforce^46^ or the forgotten patient.^47^ The present study does not corroborate these findings. Rather health‐care professionals recognized caregivers as a pillar in the care of CALD patients, and thus they became a patient proxy.

The third and final key finding suggests that caregivers of CALD patients prefer interpersonal sources of information (eg, health‐care providers). Existing research among CALD communities supports this preference,^2^, ^48^ which has been attributed to the perception that information received from knowledgeable, professional, trustworthy and respected individuals is of high value.^49^ Few caregivers in this study described the Internet as a preferred medium for information for reasons reported by other studies, including concerns about reliability.^49^, ^50^ As such, the role of information gatekeeper among caregivers of CALD patients may facilitate access to their preferred interpersonal sources, thus limiting their need to access print‐ and technology‐based sources. In contrast, caregivers of non‐CALD patients struggle to gain access to information through interpersonal sources, and consequently exhibit a higher reliance for other sources, such as the Internet to fill their knowledge gaps.^51^ Nonetheless, caregivers of CALD patients in the present study said they would still benefit from print‐based sources of information, and emphasized the importance of reviewing these materials with health‐care providers.

### 4.1 Implications for practice, research and policy

Nurses are well positioned to advocate with CALD patients and caregivers for culturally competent care, access to tailored resources, and ongoing support. This is particularly important given the high reliance of CALD communities on interpersonal sources of health information. Thus, nurses should include routine caregiver assessments into their practice^38^ to evaluate and address caregivers' perceived and unperceived needs. Further, research initiatives should include ethno‐cultural minorities to gain a better understanding of their needs and should undertake ethnographic observations of this population in situ. In particular, research should examine the interplay of language(s) and culture(s) in the provision of care.

Research should also focus on the development and evaluation of evidence‐based interventions for CALD communities that recognize the importance of addressing both patients' and caregivers' unperceived needs. Finally, tailoring existing dyadic self‐management interventions^24^ to address the specific needs of CALD communities may facilitate the acquisition and application of chronic illness self‐management skills. This involves incorporating the basic principles of health education to meet the needs of this subpopulation including: (a) in accordance with the universal recommendation in health education, a readability level at or below the fifth grade^52^; (b) vicarious learning that includes culturally and linguistically relevant examples (ie, diet, stigma, alternative medicine); (c) dissemination strategies that emphasize interpersonal contact; and (d) additional information and resources to facilitate translation and access to ethno‐specific services.

### 4.2 Limitations

Limitations of the current study include an overrepresentation of caregivers caring for patients diagnosed with chronic kidney disease (36.4%). Although a high rate of refusal (52.4%) was noted, it is lower than what has been documented in other studies (61%‐69%).^53^, ^54^ Use of phone interpreters during recruitment with CALD patients may have created a barrier to identifying eligible caregivers when compared to the success when using interpreters who were physically present during recruitment. A second limitation was the diversity of caregivers with regards to their linguistic, cultural and faith affiliations. These affiliations impact upon the migration histories of different populations, meaning that there is much diversity within the diversity captured in the category “CALD.” As such, individual diversity must be considered when applying the findings of this study to particular subgroups of CALD populations. Furthermore, the use of the term “CALD” in itself is not without limitations. Of note, is the lack of a universal definition of “CALD,” which has led scholars to criticize the term for its inability to encompass the contribution of racial differences to the differential access to social resources such as health care.^3^ Moreover, the term is said to “homogenize” the unique cultures contained within it, thereby “undermining its ability to celebrate the cultural diversity to which it lays claim”^3^ (p.7). Finally, scholars have critiqued the term CALD for lacking transparency in identifying those who are different from the majority population.^3^

A third limitation pertains to the data collection methods. Although semi‐structured interviewed were useful, they may also have resulted in certain aspects of the caregiver experiences remaining unexplored, as a result of preconceived notions of important areas warranting discussion. Furthermore, the design of the current study fails to address how the phenomenon of caring for CALD patients differs across professions, and may limit insight into the social, cultural and personal facets of caring. Finally, social desirability is a well‐documented limitation in research involving self‐reports.^55^ Therefore, caregivers may have framed their responses to present themselves favourably to the researchers.

## 5 CONCLUSION

In conclusion, health‐care providers should incorporate needs assessment tools in their practice to identify caregivers' perceived and unperceived needs; and provide information on emotional and role management aspects of caregiving. Findings of the current study contribute to the existing body of literature, and additionally provide guidance on how current caregiver interventions can be adapted to address the needs of this particular sub‐group of caregivers.

## CONFLICT OF INTEREST

We wish to confirm that there are no known conflicts of interest associated with this publication.
